# Manipulating the antioxidant capacity of halophytes to increase their cultural and economic value through saline cultivation

**DOI:** 10.1093/aobpla/plu046

**Published:** 2014-08-13

**Authors:** Christian Boestfleisch, Niko B. Wagenseil, Anne K. Buhmann, Charlotte E. Seal, Ellie Merrett Wade, Adele Muscolo, Jutta Papenbrock

**Affiliations:** 1 Institute of Botany, Leibniz University Hannover, D-30419 Hannover, Germany; 2 Seed Conservation Department, Royal Botanic Gardens Kew, Wakehurst Place, Ardingly, West Sussex RH17 6TN, UK; 3 Department of Agriculture, Mediterranea University, 89126 Reggio Calabria, Italy

**Keywords:** Halophytes, nutraceuticals, secondary metabolites, stress tolerance, yield

## Abstract

Halophytes, salt-tolerant plants, are a source of valuable secondary metabolites with potential economic value. The steady-state pools of many stress-related metabolites are already enhanced in halophytes when compared with glycophytes, but growth under conditions away from the optimum can induce stress and consequently result in changes to secondary metabolites such as antioxidants. However, direct evidence for increasing the concentration of valuable secondary metabolites as a consequence of altering the salinity of the growing environment still remains equivocal. To address this, we analysed a range of metabolites with antioxidant capacity (including total phenols, flavonoids, ascorbate, reduced/oxidized glutathione and reactive oxygen species scavenging enzymes) in seedlings and plants from different families (Amaranthaceae, Brassicaceae, Plantaginaceae and Rhizophoraceae) and habitats grown under different salt concentrations. We show that it is possible to manipulate the antioxidant capacity of plants and seedlings by altering the saline growing environment, the length of time under saline cultivation and the developmental stage. Among the species studied, the halophytes *Tripolium pannonicum*, *Plantago coronopus*, *Lepidium latifolium* and *Salicornia europaea* demonstrated the most potential as functional foods or nutraceuticals.

## Abstract (precis)

Halophytes, salt-tolerant plants, are a source of valuable secondary metabolites with potential as functional foods or nutraceuticals. We are interested in finding the optimal cultivation conditions for increasing the contents of these valuable compounds. Growth conditions away from the optimum can induce stress resulting in changes in secondary metabolites. We analyzed metabolites with antioxidant capacity in seedlings and plants from different families and habitats grown under different salt concentrations. We show that it is possible to manipulate the antioxidant capacity of plants and seedlings by altering the saline growing environment, the length of time under saline cultivation and the developmental stage.

## Introduction

The existence of spatio-temporal gradients of soil salinity has traditionally been considered one of the most important physical factors in the plant zonation of salt marshes (Chapman 1974). Zonation is caused largely by the coupling of differences in germination ecology to spatio-temporal variations in soil salinity (Egan and Ungar 2000). Salt marsh habitats are characterized by diurnal, monthly and/or seasonal dynamics where the duration of submergence, tidal scour, waterlogging and especially salinity vary (Ungar 1991). These fluctuations require high physiological plasticity, resulting in strong phenotypic and biochemical variations between individual plants, populations and species (Pigliucci *et al.* 2006; Flowers and Colmer 2008; Richards *et al.* 2010).

Biochemical perturbations in plants exposed to salinity are associated with both primary and secondary metabolisms (Král'ová *et al.* 2012) through a complex metabolic network (Krasensky and Jonak 2012; Obata and Fernie 2012), which includes an increase in intracellular levels of reactive oxygen species (ROS). Although ROS have positive roles in the stress-response pathway, for example in signalling (Kranner *et al.* 2010), an imbalance between ROS synthesis and scavenging may cause severe damage to protein structures, inhibit the activity of enzymes of important metabolic pathways and result in the oxidation of macromolecules including lipids and DNA.

Changes in the metabolic profile during exposure to salinity are dependent on the genus, species, cultivar and developmental stage of the plants (Bernstein *et al.* 2010; Atkinson and Urwin 2012). Fraire-Velázquez and Balderas-Hernández (2013) showed that the exposure of several glycophytic genera, such as *Arabidopsis*, *Lotus* and *Medicago*, to salt resulted in similar changes to 48 metabolites, with increasing concentrations of amino acids (particularly proline), sugars and phenols and with decreasing concentrations of organic acids. Conversely, halophytes do not present such large changes in metabolite profiles and/or gene expression after exposure to salt (Taji *et al.* 2004; Sanchez *et al.* 2008; Kosová *et al.* 2013), suggesting a constitutive salt tolerance. Nevertheless, environmental conditions that are sub- or supra-optimal are likely to be stressful and thus there is potential to manipulate metabolic changes through the growing environment.

Recent studies have shown the potential of halophytes as a source of valuable secondary metabolites with likely economic value (Ksouri *et al.* 2012; Buhmann and Papenbrock 2013). However, direct evidence of increases in the concentration of valuable secondary metabolites and the antioxidant capacity as a consequence of altering the salinity of the growing environment still remain equivocal. Since halophytes differ widely in their degree of salt tolerance and natural growth rate (Flowers *et al.* 1977; Ungar 1991), this study was designed to assess and compare the effect of NaCl salinity on the antioxidant capacity of halophytic species from different genera and habitats. To obtain a broad overview of their potential for functional products, several metabolites were analysed in mature plants (total phenolics, ascorbic acid (AA), total flavonoids and total antioxidant capacity), all attributed as antioxidants with use in fields such as pharmacognosy, functional foods and nutraceuticals (Arts and Hollman 2005; Scalbert *et al.* 2005; Gallie 2013). In seedlings, the antioxidant glutathione (GSH) and other low-molecular weight (LMW) thiols, and the activities of the antioxidant scavenging enzymes glutathione reductase, ascorbate peroxidase (APX), peroxidase (POX) and catalase (CAT) were evaluated. The aim was to maximize the antioxidant capacity by altering the saline growing environment and identify which of the study species have potential for cultivation as functional foods or nutraceuticals.

## Methods

### Plant material

Species were selected for study based on their habitat and taxonomy: the pioneer salt marsh plants, *Salicornia europaea*, *S. dolichostachya*, *Tripolium pannonicum* and *Suaeda maritima* (all Amaranthaceae); from the low-mid marsh, *Atriplex portulacoides* (Amaranthaceae); from the mid-upper marsh, *Atriplex halimus* (Amaranthaceae); from gravelly salty soils in dry habitats, *Plantago coronopus* (Plantaginaceae); from gravelly salty soils in wet habitats, *Lepidium latifolium* (Brassicaceae); and the woody mangrove species, *Bruguiera cylindrica* (Rhizophoraceae).

### Seeds and propagules

*Atriplex portulacoides*, *T. pannonicum* and *S. dolichostachya* seeds were collected from the North Sea coast (53°29′13″N; 8°03′16″E). *Salicornia europaea* was collected in Poland by Agnieska Piernik, Toruń. Seeds of *P. coronopus* were purchased from Jelitto Staudensamen GmbH (Schwarmstedt, Germany). One plant each of *L. latifolium* and *A. halimus* was ordered from Rühlemann's Kräuter & Duftpflanzen (Horstedt, Germany). Seeds were produced from the *L. latifolium* plant and cuttings were obtained from the *A. halimus* plant. Mature seeds of *S. maritima* were collected at Cuckmere Haven, Seaford, East Sussex in 2010.

### Germination tests

Seeds of *S. europaea* and *T. pannonicum* were selected for size homogeneity and surface sterilized for 20 min in 2.0 % (v/v) sodium hypochlorite, rinsed and soaked in distilled water for 1 h. The entire sterilization procedure including soaking took 1 h and did not affect the germination process (Ruiz-Carrasco *et al.* 2011; Burrieza *et al.* 2012). Germination tests were carried out under different concentrations of NaCl (0 mM NaCl/0 PSU, 150 mM NaCl/8.8 PSU, 300 mM NaCl/17.5 PSU and 600 mM NaCl/35.1 PSU). Seeds were placed on filter paper in 9-cm Petri dishes containing 3 mL of each solution. The Petri dishes were sealed with Parafilm to prevent evaporation and kept in a growth chamber (Green line WRS 96–85, KW, Scientific Equipment, Italy) under conditions indicated in Table 1 with a relative humidity of 70 %.

**Table 1.** Growing conditions for the plants used in the different experiments. *[see Supporting Information].

Species	Tissue	Time from sowing to salt acclimation (weeks)	Acclimation time to salt (weeks)	Time under salt treatment (weeks)	Photoperiod with additional light (h)/quantum fluence rate (µmol m^−2^ s^−1^)	Temperature (°C)	Data presented in
*L. latifolium*	Shoots	7	0	0.14	14/350	22	Fig. 1/Tables S2 and 3*
*T. pannonicum*	Shoots	3	1	5	14/350	22	Fig. 2/Table S4*
*P. coronopus*	Shoots	2	1	5	14/350	22	Fig. 3/Table S4
*T. pannonicum*	Shoots	4	1	5	14/350	22	Fig. 3/Table S4
*L. latifolium*	Shoots	4	1	5	14/350	22	Fig. 3/Table S4
*A. portulacoides*	Shoots	6	1	5	14/350	22	Fig. 3/Table S4
*A. halimus*	Shoots	7 (cuttings)	1	5	14/350	22	Fig. 3/Table S4
*S. dolicostachya*	Shoots	5	1	5	18/350	22	Fig. 3/Table S4
*B. cylindrica*	Leaves	ca. 163	6	8	14/350	22	Fig. 4/Table S4
*S. maritima*	Seedlings	0	0	<2	8/7	25	Fig. 5
*S. europaea*	Seedlings	0	0	1	16/1055	25	Table 2
*T. pannonicum*	Seedlings	0	0	1	16/1055	25	Table 3

Seeds of *S. maritima* were cleaned and stored at 15 % relative humidity and 15 °C until 1 month prior to the start of experiments when seeds were transferred to 5 °C to break their dormancy (Wetson *et al.* 2008). Seeds were sown onto two layers of 90-mm seed test paper (Fisher, UK) in 9-cm Petri dishes containing 7.5 mL of 0 mM NaCl/0 PSU, 100 mM NaCl/5.8 PSU, 200 mM NaCl/11.7 PSU, 300 mM NaCl/17.5 PSU and 400 mM NaCl/23.4 PSU. Dishes were wrapped in plastic bags and incubated at 25 °C (12 h photoperiod). Germination was defined as radicle emergence of at least 2 mm.

### Growth and cultivation of mature plants

Seeds of *L. latifolium* were germinated on propagation soil (Einheitserde, Einheitserdewerk Hameln-Tündern, Germany), and after 2 weeks transplanted to sand (0–2 mm grain size, Hornbach, Hannover, Germany), watered with modified Hoagland solution (Epstein 1972) and finally transferred to aerated containers with 13.5 L solution containing 3.57 mM NaNO_3_, 316 µM H_2_NaPO_4_ × H_2_O and 23.5 µM Fe-EDDHA (5.7 %) (Duchefa, Haarlem, Netherlands). After 1 week, the sea salt mixture was added to obtain 0 mM Na^+^ + Cl^−^/0 PSU, 220 mM Na^+^ + Cl^−^/15 PSU, 331 mM Na^+^ + Cl^−^/22.5 PSU and 442 mM Na^+^ + Cl^−^/30 PSU (Seequasal GmbH, Münster, Germany). The concentrations of the main ions were in the same range as average compositions of sea salts given in Atkinson and Bingman (1997) and are summarized in the **Supporting Information**. Both units, mM and PSU—practical salinity units, 1 PSU = 1 g sea salt kg^−1^ water—are given because many scientists in applied research use PSU. In our study, PSU was converted to mM Na^+^ + Cl^−^ not considering the other ions of the seawater, and mM NaCl was directly converted to PSU.

Seeds of *T. pannonicum*, *S. dolichostachya*, *P. coronopus*, *L. latifolium*, *A. portulacoides* were sown on propagation soil (Einheitserde). After reaching a size of ∼3 cm, the seedlings were transferred to sand (Hornbach). These seedlings and cuttings of *A. halimus* were cultivated on sand until the plants could be transferred to containers used for hydroponic culture (see Table 1 for respective time periods)*.* After a stepwise addition of 0.5 % NaCl to Hoagland solution every second day, the plants were placed in artificial seawater (Seequasal GmbH) of 220 mM Na^+^ + Cl^−^/15 PSU.

In another experiment, 4-week-old *T. pannonicum* plants, grown as described before, were transferred to containers for hydroponic culture*.* After a stepwise addition of 0.5 % NaCl to Hoagland solution every second day, the plants were placed in artificial seawater (Seequasal GmbH) of 220 mM Na^+^ + Cl^−^/15 PSU, 331 mM Na^+^ + Cl^−^/22.5 PSU and 442 mM Na^+^ + Cl^−^/30 PSU.

*Bruguiera cylindrica* propagules, originally from Indonesia, were grown in the greenhouse for ∼3 years. Plants were transferred to hydroponic basins with 110 mM Na^+^ + Cl^−^/7.5 PSU. After an acclimation time, the salinity was raised slowly and the plants were grown at salinity levels of 220 mM Na^+^ + Cl^−^/15 PSU and 442 mM Na^+^ + Cl^−^/30 PSU.

Mature plants were grown under greenhouse conditions as summarized in Table 1: sodium vapour lamps (SON-T Agro 400, Philips, Amsterdam, Netherlands) served as an additional light source, providing the minimal light flux density shown in the table. For *S. dolichostachya* day length was elongated by applying artificial light early in the morning (from 4:00 to 8:00 h) and at night (from 18:00 to 22:00 h) to prevent early flowering of the plants. Seven-week old plants were harvested at 0, 2, 4, 8 and 24 h after addition of salt.

### Determination of total flavonoids, total phenols and oxygen radical absorbance capacity (mature plants)

Unless otherwise stated, frozen ground leaf material (50 mg) of different plant species was incubated (10 min) in ice-cold methanol (800 µL, 80 %) with shaking every 2 min. After centrifugation (5 min, 15 000 *g*) the supernatant was collected and the pellet was re-extracted (three times) with ice-cold methanol (400 µL, 80 %) to produce 2 mL of extract. Extracts were used as detailed below. All reactions were performed at room temperature (RT, between 21 and 23 °C) unless stated otherwise.

#### Total phenols

Based on the method of Dudonné *et al.* (2009), 100 µL of water was pipetted into the wells of a 96-well microplate. Triplicates of 10 µL sample, blank (80 % methanol) or gallic acid standard (5–250 µg mL^−1^) were added, and finally 10 µL of Folin–Ciocalteu reagent. After incubation for 8 min and addition of 100 µL of 7 % sodium carbonate, the plate was incubated for 100 min and measured at 765 nm. Total phenols were calculated from a standard curve.

#### Total flavonoids

The method was based on Dewanto *et al.* (2002) and conducted as follows: 150 µL of water was added to each well of a clear 96-well microplate, together with 25 µL of sample or catechin hydrate standard (10–400 µg mL^−1^) or 80 % methanol as blank (in triplicate), 10 µL of 3.75 % NaNO_3_ followed by an incubation of 6 min and an addition of 15 µL of 10 % AlCl_3_. After 5 min incubation, 50 µL of 1 M NaOH was added and the total flavonoids were calculated from a standard curve based on the absorption at 510 nm.

#### Oxygen radical absorbance capacity

The oxygen radical absorbance capacity (ORAC) assay was based on Huang *et al.* (2002) and Gillespie *et al.* (2007) with modifications. A black 96-well microplate was kept on ice, 120 µL of 112 nM fluorescein in 75 mM phosphate buffer (pH 7.4) was pipetted into each well, followed by 20 µL of standards, sample or blanks. 6-Hydroxy-2,5,7,8-tetramethylchroman-2-carboxylic acid (Trolox) standard (0.25–50 µM) was diluted in the identical phosphate buffer. Samples were diluted between 1 : 40 and 1 : 150 with phosphate buffer to be within the range of the standard curve. The plate was incubated for 15 min at 37 °C and the fluorescence 485/520 was measured at time point 0. Eighty microlitres of 62 mM 2,2′-azobis(2-amidino-propane) dihydrochloride were added to each well and the fluorescence was measured every minute for 80 min. The difference in absorbance at time point 0 and after 80 min was calculated and quantified using a standard curve.

#### Ascorbic acid

The determination of AA, dehydroascorbic acid (DHA) and total ascorbic acid (TAA) was based on published protocols (Kampfenkel *et al.* 1995; Stevens *et al.* 2006; Gillespie and Ainsworth 2007). Frozen ground leaf material (50 mg) was weighed two times shaken with 500 µL of ice-cold TCA (6 %) and suspended on ice for 15 min until centrifugation (18 400 *g*, 20 min) and stored on ice before use. Cold phosphate buffer (10 µL, 75 mM, pH 7.0) and 10 µL of blank, standard (1–0.0625 mM) or sample were added to a 96-well microplate. For reduction of DHA, dithiothreitol (10 µL, 10 mM) was added to every second sample for TAA determination. After 10 min, 10 µL of *N*-ethylmaleimide (0.5 %) was added to the same second sample and 20 µL of water into the other wells. After 2 min incubation, 100 µL reaction mixture (two parts of 10 % TCA, one part of FeCl_3_, two and a half part of 43 % H_3_PO_4_ and two parts of 4 % α-α′-bipyridyl solved in 70 % ethanol) was added. After 30 min incubation at 37 °C, the absorption was read at 523 nm. The difference between the measured TAA and AA is the calculated DHA.

#### Total antioxidant capacity (seedlings)

The total antioxidant capacity was evaluated in the methanol extracts from the reduction of Mo (VI) to Mo (V) and subsequent formation of green phosphate/Mo (V) complex at acidic pH (Prieto *et al.* 1999). The extract (0.3 mL) was mixed with 3 mL of reagent solution (0.6 M sulfuric acid, 28 mM sodium phosphate and 4 mM ammonium molybdate) and the absorbance was measured at 695 nm against a blank (methanol) after cooling to RT. A calibration curve was prepared by mixing AA (31.25, 62.5, 125, 250, 500 and 1000 μg mL^−1^) with methanol and the results are expressed as mg AA equivalents g^−1^ dry matter (DM).

#### Total phenols (seedlings)

The total amount of phenols was determined using the Folin–Ciocalteu reagent (McDonald *et al.* 2001). Each plant extract (0.5 mL) was mixed with the Folin–Ciocalteu reagent (5 mL, 1 : 10 diluted with distilled water) and aqueous Na_2_CO_3_ (4 mL, 1 M). After 15 min the total phenols were determined photometrically at 765 nm. The standard curve was prepared using 0, 50, 100, 150, 200 and 250 mg L^−1^ solutions of tannic acid in methanol : water (50 : 50, v/v). Total phenol values are expressed in terms of tannic acid equivalent (mg g^–1^ DM).

#### Proline (seedlings)

The amount of proline was assayed according to Bates *et al.* (1973). Plant tissue (500 mg) was homogenized in 3 % aqueous sulfosalicylic acid (10 mL) and filtered through Whatman No. 42 filter paper. Two millilitres of acid ninhydrin (1.25 g ninhydrin in 30 mL of glacial acetic acid and 20 mL of 6 M phosphoric acid) and 2 mL of glacial acetic acid were heated for 1 h at 100 °C. The reaction mixture was extracted with 4 mL of toluene and mixed vigorously for 15–20 s and the absorbance of the toluene layer measured spectrophotometrically at 520 nm using toluene as the blank.

#### Chlorophyll and carotenoids

Frozen ground leaf material (50 mg) was extracted with ice-cold 80 % acetone (400 µL) over 10 min (with mixing every 2 min) before centrifugation at 14 000 *g* for 5 min. The supernatant was collected and stored on ice. The pellet was re-extracted three times with 200 µL ice-cold 80 % acetone and centrifuged as described. All supernatants were pooled and absorption was measured at 750.0, 663.2, 646.8 and 470.0 nm using a spectrophotometer (Uvikon XS, Biotech instruments, Germany), and total chlorophyll and carotenoid contents were calculated according to Lichtenthaler (1987).

### LMW thiols (seedlings)

For the determination of GSH, cysteine, cysteine–glycine and γ-glutamate-cysteine (reduced, oxidized and total), the plant material was freeze dried before grinding into a fine powder with a pestle and mortar using liquid nitrogen. Five replicates of 10–20 mg ground seeds were extracted in 1 mL of 0.1 M HCl and centrifuged at 15 000 *g* for 40 min at 4 °C, where the pellet was re-suspended in a further 1 mL of 0.1 M HCl, centrifuged as before and the two supernatants combined. The supernatant was used in the procedure as detailed by Seal *et al.* (2010) to determine the concentration of LMW thiols.

### Enzyme assays

#### Extracts for enzyme assays (seedlings)

Fresh seed material (0.5 g) was ground and then homogenized in 0.1 M phosphate buffer solution (pH 7.0) containing 100 mg soluble polyvinylpolypyrrolidone and 0.1 mM ethylenediaminetetraacetic acid (EDTA). The homogenates were centrifuged at 15 000 *g* for 15 min at 4 °C and the resulting supernatants filtered through two layers of muslin cloth and used for enzyme activity assays. All enzyme activities were measured at 25 °C in a UV–visible light spectrophotometer (UV-1800 CE, Shimadzu, Japan) and expressed as enzyme units (U) per milligram soluble protein where one unit of enzyme was defined as the amount of enzyme necessary to decompose 1 µmol of substrate per min at 25 °C.

#### Guaiacol peroxidase

Peroxidase (EC 1.11.1.7) activity was measured following the change in absorbance at 430 nm during the incubation of the extracts at 25 °C with 10 mM guaiacol, 1 mM H_2_O_2_ in 0.1 M potassium phosphate buffer at pH 7.0 in a total volume of 3 mL. The molar extinction coefficient was 2.55 mM^−1^ cm^−1^ (Putter 1974).

#### Ascorbate peroxidase

Ascorbate peroxidase (EC 1.11.1.11) activity was assayed according to Nakano and Asada (1981). The reaction mixture (1.5 mL) contained 50 mM phosphate buffer (pH 6.0), 0.1 μM EDTA, 0.5 mM ascorbate, 1.0 mM H_2_O_2_ and 50 μL enzyme extract. The reaction was started by the addition of H_2_O_2_ and ascorbate oxidation measured at 290 nm for 1 min. Enzyme activity was quantified using the molar extinction coefficient for ascorbate (2.8 mM^−1^ cm^−1^) and the results were expressed in μmol H_2_O_2_ min^−1^ mg^−1^ protein taking into consideration that 2 mol of ascorbate are required for the reduction of 1 mol H_2_O_2_ (McKersie and Leshem 1994).

#### Catalase

Catalase (EC 1.11.1.6) activity was measured according to the method of Beers and Sizer (1952), with minor modifications. The reaction mixture (1.5 mL) consisted of 100 mM phosphate buffer (pH 7.0), 0.1 μM EDTA, 20 mM H_2_O_2_ and 50 μL enzyme extract. The reaction was started by the addition of the extract. The decrease of H_2_O_2_ was monitored at 240 nm and quantified by its molar extinction coefficient (36 mM^−1^ cm^−1^) and the results were expressed as μmol H_2_O_2_ min^−1^ mg^−1^ protein.

#### Glutathione reductase

Glutathione reductase (EC 1.6.4.2) activity was determined by measuring the rate of NADPH_2_ oxidation as the decrease in absorbance at 340 nm (*ɛ* = 6.2 mM^−1^ cm^−1^). The reaction mixture contained 0.1 M potassium phosphate buffer (pH 7.0), 20 mM GSSG, 2 mM NADPH_2_, 350 µL H_2_O and 50 µL enzyme extract.

### Statistical analysis

#### Mature plants

Four plants (shoots including leaves) were used at each data point in triplicate for ORAC, total phenols and total flavonoids, and in duplicate for AA. Values were tested for significance with a two-way analysis of variance (ANOVA) and Holm-Sidak post-hoc analysis using SigmaPlot 12.5 (Systat Software, Inc.). The grouping factors were salinity and time; interactions were calculated for ORAC, TAA, phenols and flavonoid values.

Three plants (shoots including leaves) were pooled as one sample, and three (one, as starting value for the species comparison) pooled samples made one data point except for *B. cylindrica* where eight to nine leaves were analysed individually. The analysis was repeated three times in triplicate measurements for ORAC, total phenols and total flavonoids, and in duplicate for AA. Carotenoids and chlorophyll were measured three times. Results were tested for significance by one-way ANOVA and *B. cylindrica* by *t*-test in SigmaPlot 12.5 (Systat Software, Inc.).

#### Seedlings

For experiments with *S. maritima*, five replicates of 120 seeds were used. Reduced GSH and glutathione disulfide (GSSH) values were tested for significance with one-way ANOVA (*P* > 0.05). Germination values were arcsine transformed before tested with one-way ANOVA and post-hoc analysis of LSD (*P* < 0.05).

For experiments with *S. europaea* and *T. pannonicum* seedlings, five replicates of 50 seeds were used. All data were analysed by one-way ANOVA with the salt concentration as the grouping factor. The response variables for these ANOVAs were seed germination, total antioxidant activity, total phenol and enzyme activities. Since salt concentration had four levels, on all significant ANOVAs we performed the Tukey multiple comparison test to compare all pairs of means. The germination percentage data were previously subjected to arcsine transformation and were reported in tables as untransformed values. All data collected were statistically analysed using SYSTAT 8.0 software (SPSS, Inc.).

## Results

### Antioxidant capacity of plants at different salinities

#### Lepidium latifolium

**Figure 1.**
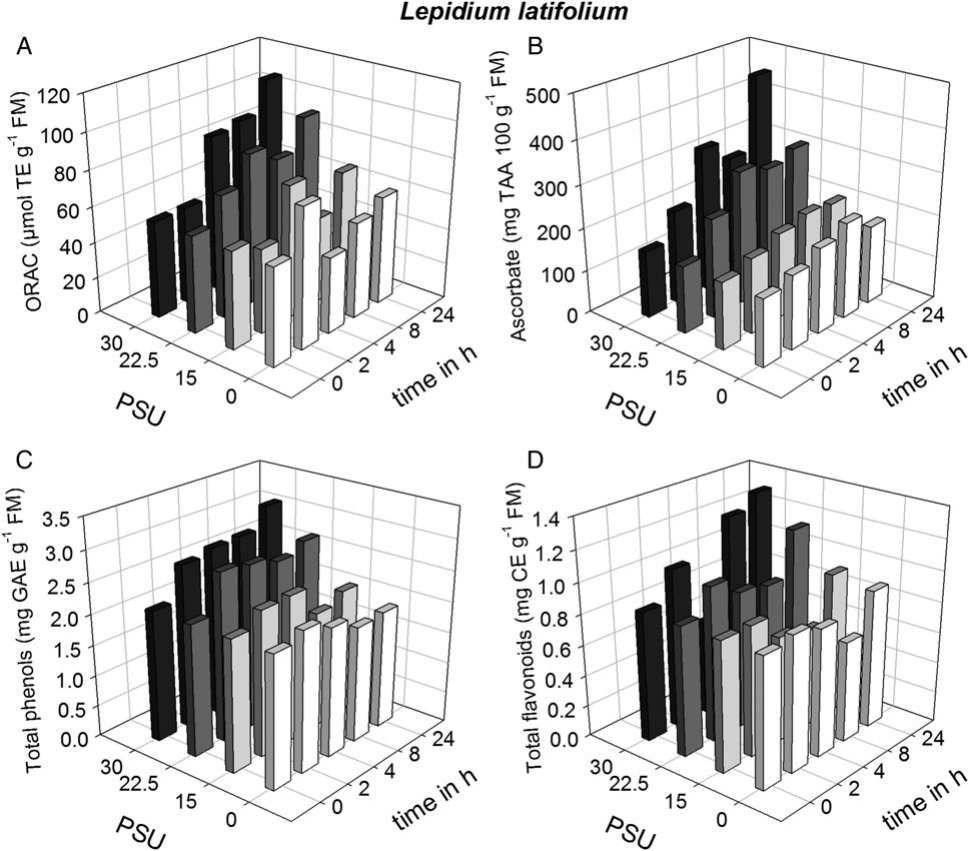
Six-week-old *L. latifolium* plants were transferred to basins; after 1 week the salinity was increased directly to 220 mM Na^+^ + Cl^−^/15 PSU, 331 mM Na^+^ + Cl^−^/22.5 PSU and 442 mM Na^+^ + Cl^−^/30 PSU. Samples were taken at time point 0 just before increasing salinity and 2, 4, 8, 24 h thereafter. Means are plotted against PSU and time. (A) ORAC values, (B) ascorbate, (C) total phenols and (D) total flavonoids. CE, catechin equivalents; GAE, gallic acid equivalents; TAA, total ascorbic acid; TE, trolox equivalents. For statistical analysis see **Supporting Information**.

The aim was to analyse the effect of different salt concentrations on the antioxidant capacity of different halophytic species. Oxygen radical absorbance capacity values of 7-week-old *L. latifolium* plants in the control and at 220 mM Na^+^ + Cl^−^/15 PSU did not change significantly during the time course of the experiment (Fig. 1A, **[see Supporting Information]**). However, the higher salt concentrations (331 mM Na^+^ + Cl^−^/22.5 PSU and 442 mM Na^+^ + Cl^−^/30 PSU) led to significant increases in the ORAC values (Holm-Sidak method, *P* < 0.05). After 24 h, the values were approximately double of that at the beginning of the experiment. In parallel with changes in the ORAC values, the concentration of DHA and TAA increased significantly at higher salinities (Fig. 1B, *P* < 0.05). Also an increase of total phenols was observed in control plants (up to 24 h) and at 220 mM Na^+^ + Cl^−^/15 PSU (up to 8 h) (Fig. 1C) but fluctuated at higher salt concentrations. The flavonoid concentrations were almost constant in the control and at 220 mM Na^+^ + Cl^−^/15 PSU but increased at the higher at 331 mM Na^+^ + Cl^−^/22.5 PSU and 442 mM Na^+^ + Cl^−^/30 PSU (Fig. 1D). The grouping factors were salinity and time. For TAA and flavonoid values, there are significant interactions between salinity and time, whereas for phenol and ORAC values there are no significant interactions between time and salinity. For comparison the values are also expressed on a dry matter basis **[see Supporting Information]**. In summary, in *L. latifolium* increasing salt concentrations induced a higher antioxidative capacity based on TAA, total phenols and/or flavonoids.

#### Tripolium pannonicum

**Figure 2.**
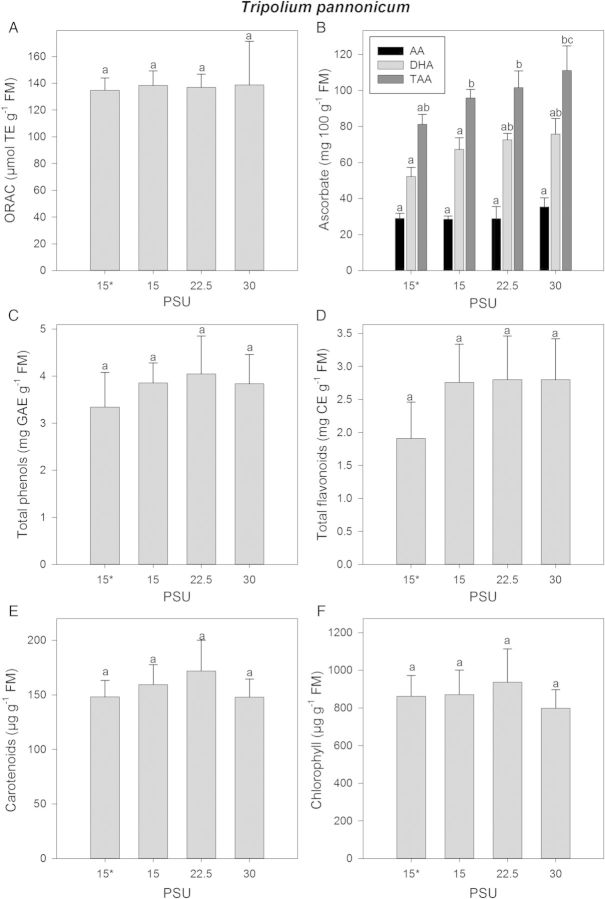
*Tripolium pannonicum* plants were acclimated to a salinity of 220 mM Na^+^ + Cl^−^/15 PSU for 1 week and thereafter transferred to basins of 220 mM Na^+^ + Cl^−^/15 PSU, 331 mM Na^+^ + Cl^−^/22.5 PSU and 442 mM Na^+^ + Cl^−^/30 PSU. Plants taken after the salt acclimation are indicated with an asterisk. Each sample was analysed for (A) ORAC values, (B) AA, DHA and TAA, (C) total phenols, (D) total flavonoids, (E) carotenoid and (F) chlorophyll contents. For abbreviations see legend to Fig. 1. Different letters indicate significant differences.

The antioxidant capacity of 9-week-old salt-adapted *T. pannonicum* plants did not show any significant differences from the control (4-week-old plants) at different salinities (Fig. 2A). The amounts of AA, DHA and TAA increased slightly with increasing salinity but this was not significant (Fig. 2B). The concentrations of total phenols and total flavonoids increased when the plants were grown in the presence of salt, although different salt concentrations in the medium did not increase the concentrations significantly (Fig. 2C and D). The carotenoid (Fig. 2E) and chlorophyll (Fig. 2F) concentrations increased with increasing salinity until 331 mM Na^+^ + Cl^−^/22.5 PSU, after which by 442 mM Na^+^ + Cl^−^/30 PSU the values were at their lowest. Taken together in salt-adapted mature *T. pannonicum*, increasing salt concentrations did not induce a higher antioxidative capacity although there was a slight increase in potential antioxidants, such as phenols, flavonoids and carotenoids.

#### Atriplex halimus, A. portulacoides, L. latifolium, P. coronopus, S. dolichostachya, T. pannonicum

**Figure 3.**
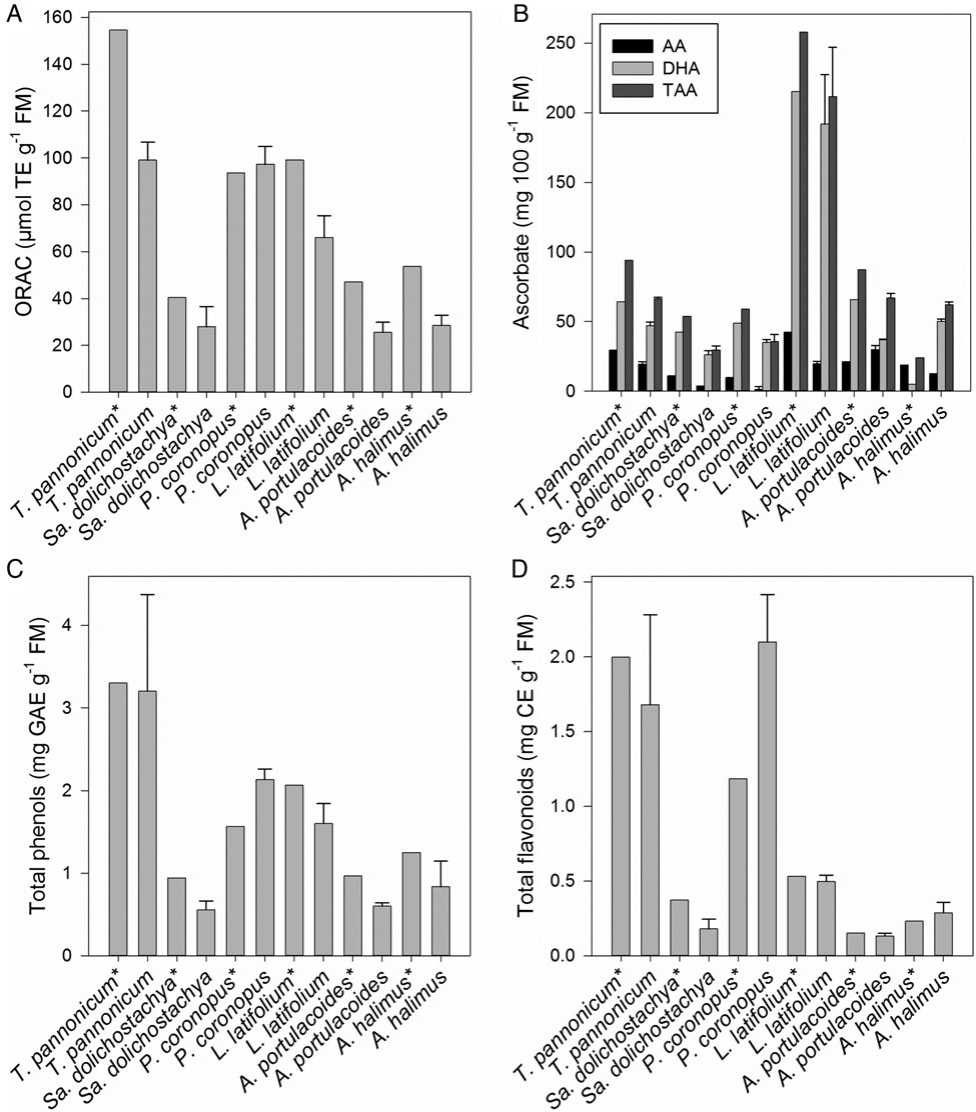
Different halophyte species, *T. pannonicum*, *S. dolichostachya*, *P. coronopus*, *L. latifolium*, *A. portulacoides* and *A. halimus*, were grown on sand for up to 6 weeks, acclimated for 1 week to a salinity of 220 mM Na^+^ + Cl^−^/15 PSU and transferred to basins containing 220 mM Na^+^ + Cl^−^/15 PSU. One sample was taken at the beginning of the experiment (marked with an asterisk). After 5 weeks samples were taken again. Each sample was analysed for (A) ORAC values, (B) AA, DHA and total ascorbic acid (TAA), (C) total phenols and (D) total flavonoids. For abbreviations see legend to Fig. 1. Different letters indicate significant differences.

In a comparison of different halophytic species, the antioxidant capacities were determined among young, non-treated plants and 7-week-old and 11-week-old plants grown at 220 mM Na^+^ + Cl^−^/15 PSU (Fig. 3). The ORAC showed significant differences among the salt-tolerant species (Fig. 3A). With the exception of *P. coronopus*, the ORAC values were always lower in the older, salt-treated plants. The amount of DHA and therefore the TAA amounts rose with increasing salinity (Fig. 3B).Younger and unstressed halophytes contained higher concentrations of TAA except *A. halimus* which showed a large increase in DHA. The higher ORAC values in *P. coronopus* were based on higher contents of total phenols and flavonoids whereas in the other plant species investigated the levels were lower in older, salt-treated plants (Fig. 3C and D). On the one hand, younger plants have higher concentrations of antioxidant compounds, on the other hand are much smaller and have less biomass.

#### Bruguiera cylindrica

**Figure 4.**
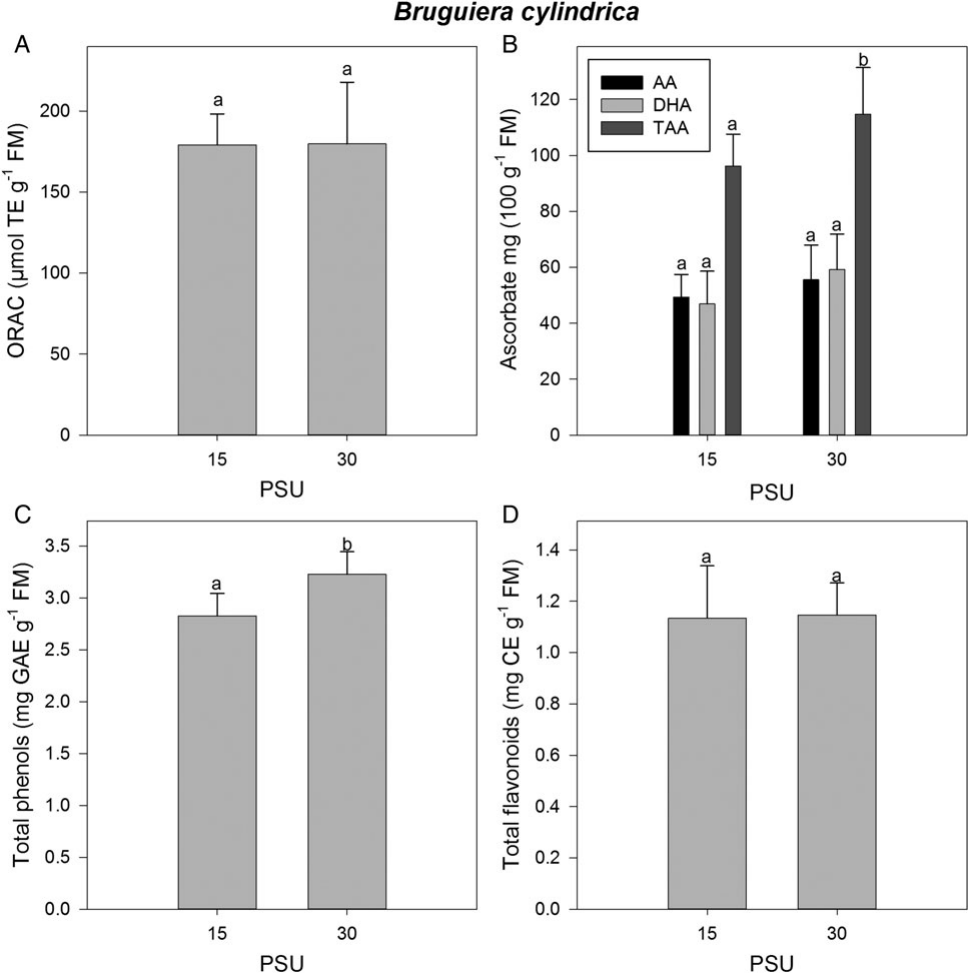
*Bruguiera cylindrica* plants were transferred to basins with 110 mM Na^+^ + Cl^−^/7.5 PSU. After an acclimation time of 7 weeks the salinity was raised slowly over 6 weeks and the plants were grown 8 weeks at salinity levels of 220 mM Na^+^ + Cl^−^/15 PSU and 442 mM Na^+^ + Cl^−^/30 PSU. Leaf material was harvested. Each sample was analysed for (A) ORAC values, (B) AA, DHA and TAA, (C) total phenols and (D) total flavonoids. For abbreviations see legend to Fig. 1. Different letters indicate significant differences.

The ORAC values were not influenced by doubling the salt concentration in the growth medium in *B. cylindrica* trees (Fig. 4A). However, AA, DHA and TAA as well as total phenols and flavonoid concentrations were increased at higher salt concentrations (Fig. 4B–D) indicating a huge source of antioxidant compounds.

### Antioxidant capacity of seedlings at different salinities

#### Suaeda maritima

**Figure 5.**
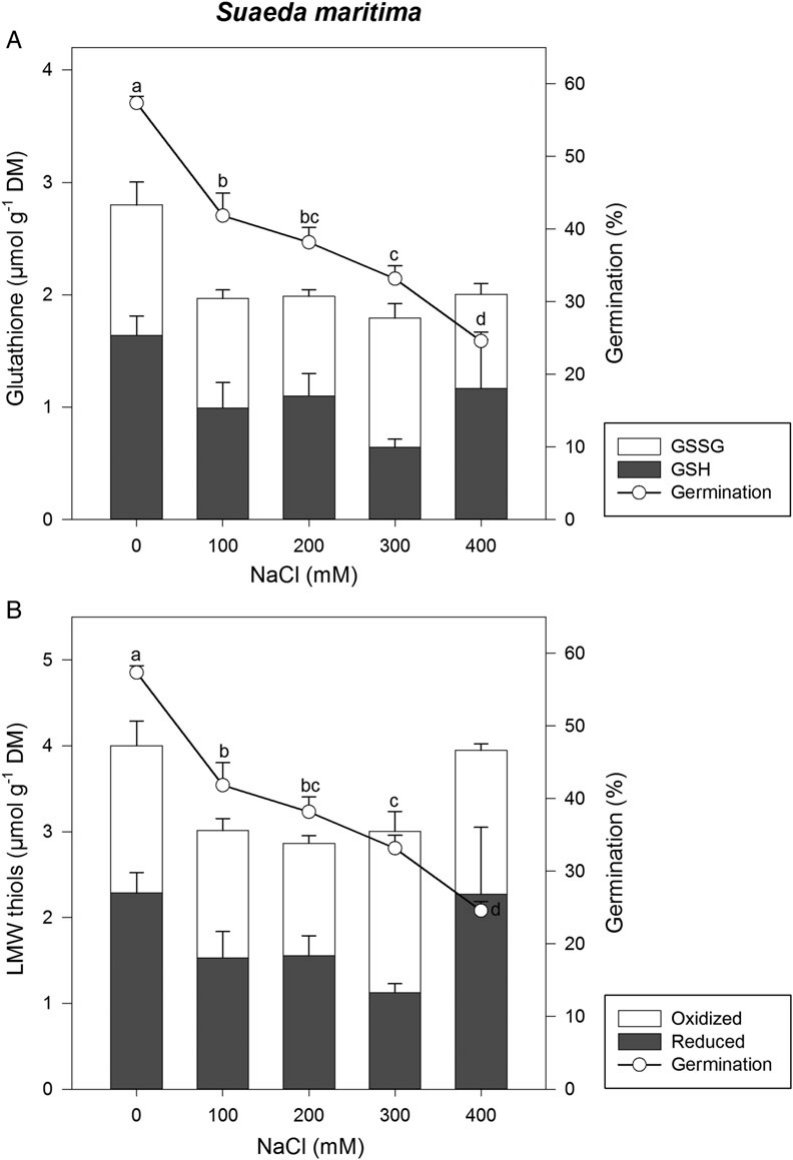
(A) Reduced GSH (grey bars) and GSSG (white bars) in germinated seeds of *S. maritima*. Line plot indicates viability of seeds as determined by germination test. Germination values are shown as mean ± SE. Different letters indicate significant differences of the mean. (B) Reduced concentration of the LMW thiols glutathione + cysteine + cysteinyl–glycine + γ-glutamyl-cysteinyl (grey bars) and their oxidized forms (white bars) in germinated seeds of *S. maritima*. No significant differences in the concentration of LMW thiols (reduced, oxidized and total) were seen with increasing NaCl. Line plot indicates viability of seeds as determined by germination test. Germination values are shown as mean values ± SE. Different letters indicate significant differences of the mean.

The germination of *S. maritima* was highest in the absence of NaCl (58 %) and decreased with increasing NaCl concentration to 24 % at 400 mM NaCl/23.4 PSU (Fig. 5A and B). Although there were small changes in the concentration of GSH (Fig. 5A) and LMW thiols (Fig. 5B) with increasing NaCl treatment, these were not significant (*P* > 0.05). No significant differences in the concentration of GSH, GSSG or total glutathione were seen with increasing NaCl. The ratio of GSH : GSSG was similar at all salt treatments, with a slightly higher proportion of reduced than oxidized glutathione (ratio GSH : GSSG between 1.02 and 1.42), except at 300 mM NaCl/17.5 PSU where oxidized GSSG was dominant (ratio GSH : GSSG 0.56). The ratio of reduced : oxidized thiols was also similar at all salt treatments, with a slightly higher proportion of reduced than oxidized thiols (ratio LMW thiols reduced : oxidized between 1.03 and 1.35), except at 300 mM NaCl/17.5 PSU where oxidized thiols were dominant (ratio LMW thiols reduced : oxidized 0.60).

#### Salicornia europaea

The germination of *S. europaea* did not change with increasing salinity from 0 to 300 mM NaCl/17.5 PSU; however, an increase in the total antioxidant capacity, APX, CAT, GR, proline and phenols was observed (Table 2). At the highest salinity (600 mM NaCl/35.1 PSU), a significant decrease in germination (from 85 to 50 %) in comparison with the other treatments was observed while all other metabolites measured, except proline, increased to their highest levels (Table 2).

**Table 2.** Total antioxidant capacity, proline content and activities of guaiacol peroxidase, CAT, APX and glutathione reductase enzymes in 7-day-old seedlings of *S. europaea* germinated under different NaCl treatments. Values are means ± SE (*n* = 4). Different letters in the same column indicate significant differences *P* ≤ 0.01. AAE, ascorbic acid equivalents.

NaCl treatments	Germination percentage (%)	Total antioxidant capacity (AAE g^−1^ DM)	POX (U mg^−1^ prot)	APX (U mg^−1^ prot)	CAT (U mg^−1^ prot)	GR (U mg^−1^ prot)	Proline (mg g^−1^ DM)	Phenols (mg tannic acid g^−1^ DM)
0 mM NaCl/0 PSU	85^a^	32.74 ± 1.11^c^	0.26 ± 0.1^b^	1.41 ± 0.5^c^	25 ± 2.0^c^	0.05 ± 0.03^d^	0.33 ± 0.03^b^	237.9 ± 5.0^c^
150 mM NaCl/8.8 PSU	85^a^	44.51 ± 3.76^b^	0.29 ± 0.2^b^	1.74 ± 0.4^c^	24 ± 2.1^c^	0.11 ± 0.02^c^	0.59 ± 0.02^a^	445.9 ± 4.0^b^
300 mM NaCl/17.5 PSU	85^a^	47.93 ± 3.11^b^	0.56 ± 0.3^b^	2.52 ± 0.2^b^	29 ± 2.5^b^	0.16 ± 0.02^b^	0.62 ± 0.03^a^	437.9 ± 4.8^b^
600 mM NaCl/35.1 PSU	50^b^	58.96 ± 4.21^a^	1.1 ± 0.2^a^	3.16 ± 0.3^a^	37 ± 3.1^a^	0.20 ± 0.01^a^	0.61 ± 0.02^a^	675.3 ± 5.5^a^

#### Tripolium pannonicum

Seeds of *T. pannonicum* germinated in the presence of 150 mM NaCl/8.8 PSU to the same extent as at 0 mM NaCl but increases in CAT, GR and phenols were apparent (Table 3). At 300 mM NaCl/17.5 PSU germination decreased to 50 % together with a significant increase in total antioxidant capacity, POX, APX, CAT, GR, proline and phenols (Table 3). No germination was observed at the highest salinity (600 mM Na Cl/35.1 PSU).

**Table 3.** Total antioxidant capacity, proline content and activities of guaiacol peroxidase, CAT, APX and glutathione reductase enzymes in 7-day-old seedlings of *T. pannonicum* germinated under different NaCl treatments. Values are means ± SE (*n* = 4). Different letters in the same column indicate significant differences *P* ≤ 0.01. AAE, ascorbic acid equivalents.

NaCl treatments	Germination percentage (%)	Total antioxidant capacity (AAE g^−1^ DM)	POX (U mg^−1^ prot)	APX (U mg^−1^ prot)	CAT (U mg^−1^ prot)	GR (U mg^−1^ prot)	Proline (mg g^−1^ DM)	Phenols (mg tannic acid g^−1^ DM)
0 mM NaCl/0 PSU	91 ± 2.1^a^	18.15 ± 1.61^b^	0.29 ± 0.21^b^	1.11 ± 0.4^b^	20 ± 2.2^c^	0.12 ± 0.02^c^	0.41 ± 0.14^b^	237.9 ± 5.0^c^
150 mM NaCl/8.8 PSU	88 ± 3.3^a^	23.33 ± 3.76^b^	0.67 ± 0.33^b^	2.04 ± 0.6^b^	31 ± 3.5^b^	0.27 ± 0.01^b^	0.60 ± 0.19^b^	345.9 ± 6.0^b^
300 mM NaCl/17.5 PSU	50^b^	39.44 ± 3.11^a^	1.68 ± 0.41^a^	3.99 ± 0.4^a^	53 ± 4.1^a^	0.43 ± 0.01^a^	1.09 ± 0.21^a^	437.9 ± 8.0^a^
600 mM NaCl/35.1 PSU	0	0	0	0	0	0	0	0

## Discussion

Antioxidants such as phenolic compounds, carotenoids, AA and flavonoids are ubiquitous in plants and are essential to the human diet (Arrigoni and De Tullio 2002; Balasundaram *et al.* 2006; Pham-Huy *et al.* 2008; Parvaiz *et al.* 2009; Ksouri *et al.* 2012; Fiedor and Burda 2014). The association of a raised antioxidant capacity with salt tolerance has been demonstrated in a number of salt-tolerant glycophytes and true halophytes (Broetto *et al.* 2002; Agarwal and Pandey 2004; Ben Amor *et al.* 2005; Alhdad *et al.* 2013). When salinity was applied over 24 h to plants of the annual halophyte *L. latifolium*, an increase in total antioxidant capacity in addition to phenols, ascorbate and flavonoids was observed, demonstrating that manipulation of the antioxidant capacity is possible through salinity treatment. Furthermore, the TAA contents of *L. latifolium* were higher than in the other halophyte species studied (Fig. 3B) and higher than the reported values in glycophytes within this genus such as *Lepidium sativum* (garden cress; 60 mg 100 g^−1^ FM, http://ndb.nal.usda.gov/ndb/search/list, accessed 29 March 2014) and cabbage (90–150 mg 100 g^−1^ FM), and species outside the genus such as the lemon fruit (*Citrus* × *limon*; 53 mg 100 g^−1^ FM, http://ndb.nal.usda.gov/ndb/search/list, accessed 29 March 2014). Therefore, the treatment of *L. latifolium* with high salt concentration for 24 h could significantly increase the ascorbate contents and could be used as a promising cultivating technique.

In contrast to the 24 h treatment of *L. latifolium*, mature plants of *T. pannonicum* did not accumulate secondary compounds when exposed to 15–30 PSU salinity for 4 weeks (Fig. 2). For many of the mature plant species studied, and in seedlings of *S. maritima*, the antioxidant capacity remained similar after weeks of salt exposure (Figs. 3–5). This could indicate that the levels of salinity were near optimal and therefore not high enough to induce a stress response or reflect a period where the antioxidant mechanisms have adjusted or ‘resisted’ (*sensu* Kranner *et al.* 2010) to the increasing stress. For other species, the antioxidant capacity decreased after 5 weeks salt exposure (Fig. 3), suggesting that increased exposure time to salinity causes failure in these protective mechanisms, which in turn leads to a loss of vigour and eventually death of the organism (Kranner *et al.* 2010; Kranner and Seal 2013). According to the triphasic stress concept of Kranner *et al.* (2010), the activation of protection and repair machinery, which includes the antioxidant defence system, occurs soon after stress is perceived at the cellular level (e.g. by changes in osmotic potential or ionic homoeostasis) and could explain the more noticeable increase in antioxidant capacity under the shorter 24-h salinity exposure period in *L. latifolium*.

Whereas 4- and 5-week-old plants of *T. pannonicum* had little change in total antioxidant, 7-day-old seedlings did (Table 3) in addition to seedlings of *S. europaea*. Cultivated for 1 week, increasing salinity was associated with a higher antioxidant capacity, particularly with respect to proline and total phenols, and the ROS-scavenging enzyme activities. Proline is an osmoprotectant and a low-molecular-weight chaperone, and can reduce the inhibitory effects of ions on enzyme activity, increase the thermal stability of enzymes and prevent the dissociation of enzyme complexes such as the oxygen-evolving complex of PSII (Hasegawa *et al.* 2000). Furthermore, the enzymes required for pathway extensions that lead to these osmolytes are often induced following salt and drought stresses (Hasegawa *et al.* 2000; Hong Bo *et al.* 2006). Many comparative studies using salt-tolerant and sensitive genotypes have correlated higher salt tolerance to an increase in the activity of antioxidant enzymes (Crowe *et al.* 1984; Ashraf 1994; Ashraf and Fatima 1995; Garcia *et al.* 1997; Hounsa *et al.* 1998). Overexpression of some antioxidant enzymes has been reported to improve salt tolerance (Singer and Lindquist 1998; Goddijn and van Dun 1999).

Other environmental factors such as light intensity should be considered. The mangrove *B. cylindrica* did contain higher phenols after salinity exposure but not flavonoid contents despite the literature reporting that mangroves use phenols and tannins as a UV protection screen (Moorthy and Kathiresan 1997). It is likely that higher light intensities in parallel with higher salt concentrations are necessary to raise the antioxidant capacity in this mangrove species. *Tripolium pannonicum* showed a maximum of carotenoid and chlorophyll contents at 331 mM NaCl/22.5 PSU. If high concentrations of photosynthetic pigments are taken as an indication for high photosynthetic activity and consequently for increased growth and biomass production, higher salinities were favourable in comparison to lower salt concentration. However, in the case of *T. pannonicum* a lower yield in biomass production was correlated with increasing salinity. The gain in fresh weight biomass per plant during 5 weeks was 51.4 ± 12.8 g at 220 mM Na^+^ + Cl^−^/15 PSU, 27.5 ± 5.6 g at 331 mM Na^+^ + Cl^−^/22.5 PSU and 14.6 ± 2.3 g at 442 mM Na^+^ + Cl^−^/30 PSU.

The manipulation of cultivation conditions to increase antioxidant capacity is only desirable if plant growth does not decline. For example, treatment of *L. latifolium* with 15 PSU salinity (equivalent to 220 mM Na^+^ + Cl^−^) resulted in the highest biomass production among the treatments (data not shown). Previously, plants of *S. maritima* with higher antioxidant capacity induced during saline flooding were associated with a biomass yield penalty in plants grown in both the natural environment and glasshouse (Alhdad *et al.* 2013). Furthermore, an increase in phenols and flavonoids was observed in *Nitraria retusa* and *A. halimus* with increasing salinity but this was associated with decreased growth at concentrations of NaCl between 400 mM/23.4 PSU and 800 mM/46.8 PSU (Boughalleb and Denden 2011). A balance is therefore needed so that the antioxidant capacity is enhanced from salinity treatment and growth is maintained. The risk of cultivating halophytes under saline conditions too distant from the optimum environment is that energy resources may be directed into additional energy-demanding stress-response mechanisms, such as ion transport (Flowers and Colmer 2008).

## Conclusion

This study has directly shown that it is possible to manipulate the antioxidant capacity of plants and seedlings by altering the saline growing environment. The length of time cultivated under saline conditions and the developmental stage may influence the metabolite levels. Seedlings of the obligate halophyte *S. europaea* are good candidates for antioxidant manipulation particularly as these plants are already established in the market as desirable edible crops. Mature plants of *L. latifolium* exposed to short-term salinity may also be a good candidate, especially as a source for AA. Among the species studied, the total antioxidant capacity in the halophytes *T. pannonicum*, *P. coronopus* and *L. latifolium* cultivated under moderate salt stress is higher than fruits and vegetables generally sold in the market (Ninfali *et al.* 2005), demonstrating the potential of these halophytes as functional foods or nutraceuticals.

## Sources of Funding

The research in the Hannover laboratory and travelling was funded by the Deutsche Bundesstiftung Umwelt (AZ 27708) and by COST (STSM FA0901-041011-011415). The Royal Botanic Gardens, Kew, receive grant in aid from DEFRA.

## Contributions by the Authors

C.B., N.B.W., A.K.B. and E.M.W. conducted the experimental work and analysed the data. C.E.S., A.M. and J.P. analysed the data and wrote the manuscript.

## Conflicts of Interest Statement

None declared.

## Supporting Information

The following Supporting Information is available in the online version of this article –

**Table S1.** Mean molarity values from eight commercially available sea salts calculated for three different salinity concentrations. Data from Atkinson and Bingman (1997).

**Table S2.** Mean values and SD (*n* = 4) for *L. latifolium* fresh matter (FM)*.* See Fig. 1 for further details. Different letters at the same time point indicate significant differences (*P* ≤ 0.05). All pairwise multiple comparison procedures (Holm-Sidak method) were applied. AA, ascorbic acid; CE, catechin equivalents; DHA, dehydroascorbic acid; GAE, gallic acid equivalents; TAA, total ascorbic acid; TE, trolox equivalents.

**Table S3.** Mean values and SD (*n* = 4) for *L. latifolium* dry matter (DM)*.* See Fig. 1 for further details. Different letters at the same time point indicate significant differences (*P* ≤ 0.05). All pairwise multiple comparison procedure (Holm-Sidak method) was applied. AA, ascorbic acid; CE, catechin equivalents; DHA, dehydroascorbic acid; GAE, gallic acid equivalents; TAA, total ascorbic acid; TE, trolox equivalents.

**Table S4.** Glutathione and cysteine concentrations of different species corresponding to different salinities. The asterisk marks the start values of younger plants *^1^4 weeks, *^2^up to 7 weeks old. These plants were harvested just after the adaptation to 220 mM Na^+^ + Cl^−^/15 PSU.

Additional Information
