# Supplementary material for: Manipulating the antioxidant capacity of halophytes to increase their cultural and economic value through saline cultivation
Source: AoB Plants. 2014 Aug 13;6:plu046. doi: 10.1093/aobpla/plu046 (PMC4174659; doi:10.1093/aobpla/plu046)
Supplement: Additional Information [file supp_6_plu046_index.html]

Manipulating the antioxidant capacity of halophytes to increase their cultural and economic value through saline cultivation — Manipulating the antioxidant capacity of halophytes to increase their cultural and economic value through saline cultivation — Additional Information 

# Manipulating the antioxidant capacity of halophytes to increase their cultural and economic value through saline cultivation

## Additional Information

Additional Information

**Files in this Data Supplement:**

- Additional Information File 1 - docx file
- Additional Information File 2 - docx file
- Additional Information File 3 - docx file
- Additional Information File 4 - docx file
