# Supplementary material for: Manipulating the antioxidant capacity of halophytes to increase their cultural and economic value through saline cultivation
Source: AoB Plants. 2014 Aug 13;6:plu046. doi: 10.1093/aobpla/plu046 (PMC4174659; doi:10.1093/aobpla/plu046)
Supplement: Additional Information [file supp_plu046_plu046supp_file1.docx]

**File 1**. Mean molarity values from eight commercially available sea salts calculated for three different salinity concentrations. Data from Atkinson and Bingman (1997).

| **Major cations (mM)** | **15 PSU** | **22.5 PSU** | **30 PSU** |
| --- | --- | --- | --- |
| Na^+^ | 199.9 | 299.9 | 399.9 |
| K^+^ | 4.184 | 6.276 | 8.368 |
| Mg^2+^ | 22.13 | 33.19 | 44.25 |
| Ca^2+^ | 4.184 | 6.276 | 8.368 |
| Sr^+^ | 0.053 | 0.080 | 0.106 |
| **Major anions (mM)** |  |  |  |
| Cl^-^ | 226.4 | 339.6 | 452.8 |
| SO_4_^2-^ | 10.71 | 16.07 | 21.43 |
| **Nutrients (µM)** |  |  |  |
| PO_4_-P | 0.219 | 0.328 | 0.437 |
| NO_3_-N | 2.002 | 3.003 | 4.004 |
| NH_4_-N | 2.890 | 4.335 | 5.780 |
