# Supplementary material for: Manipulating the antioxidant capacity of halophytes to increase their cultural and economic value through saline cultivation
Source: AoB Plants. 2014 Aug 13;6:plu046. doi: 10.1093/aobpla/plu046 (PMC4174659; doi:10.1093/aobpla/plu046)
Supplement: Additional Information [file supp_plu046_plu046supp_file2.docx]

**File 2.** Mean values and SD (n=4) for *L. latifolium* fresh matter (FM)*.* See Figure 1 for further details. Different letters at the same time point indicate significant differences (P≤0.05). All Pairwise Multiple Comparison Procedure (Holm Sidak Method) was applied. Abbreviations: Ascorbic acid, AA; catechin equivalents, CE; dehydroascorbic acid, DHA; gallic acid equivalents, GAE; total ascorbic acid, TAA; trolox equivalents, TE.

| **PSU** | **mM Na^+^ + Cl^-^** | **Time point** | **ORAC (µmol TE g^-1^ FM)** | | **AA (mg 100 g^-1^ FM)** | | **DHA (mg 100 g^-1^ FM)** | | **TAA (mg 100 g^-1^ FM)** | | **Total phenols (mg GAE g^-1^ FM)** | | **Total flavonoids (mg CE g^-1^ FM)** | |
| --- | --- | --- | --- | --- | --- | --- | --- | --- | --- | --- | --- | --- | --- | --- |
| 0 | 0 | 0 | 54.44 | ±31.24^a^ | 36.53 | ±5.06^a^ | 120.7 | ±22.33^a^ | 157.2 | ±20.92^a^ | 2.129 | ±0.506^a^ | 0.843 | ±0.220^a^ |
| 0 | 0 | 2 | 77.86 | ±16.66^a^ | 39.05 | ±7.81^a^ | 134.1 | ±20.06^a^ | 173.2 | ±27.05^a^ | 2.259 | ±0.459^a^ | 0.877 | ±0.164^a^ |
| 0 | 0 | 4 | 42.66 | ±8.29^a^ | 39.35 | ±3.65^a^ | 160.8 | ±44.72^a^ | 200.1 | ±45.34^a^ | 2.085 | ±0.149^a^ | 0.827 | ±0.048^a^ |
| 0 | 0 | 8 | 53.92 | ±18.35^a^ | 38.71 | ±2.41^a^ | 186.0 | ±23.10^a^ | 224.7 | ±24.47^ac^ | 1.876 | ±0.243^a^ | 0.654 | ±0.243^a^ |
| 0 | 0 | 24 | 60.78 | ±24.44^a^ | 26.23 | ±4.30^a^ | 158.4 | ±18.83^a^ | 184.6 | ±21.46^a^ | 1.908 | ±0.338^a^ | 0.900 | ±0.106^a^ |
| 15 | 220 | 0 | 54.44 | ±31.24^a^ | 36.53 | ±5.06^a^ | 120.7 | ±22.33^a^ | 157.2 | ±20.92^a^ | 2.129 | ±0.506^a^ | 0.843 | ±0.220^a^ |
| 15 | 220 | 2 | 47.31 | ±21.12^a^ | 43.13 | ±11.39^a^ | 133.2 | ±36.84^a^ | 176.3 | ±25.84^a^ | 2.351 | ±0.354^a^ | 0.847 | ±0.120^a^ |
| 15 | 220 | 4 | 74.41 | ±30.20^a^ | 45.56 | ±13.12^a^ | 154.7 | ±50.77^a^ | 200.3 | ±45.81^a^ | 2.371 | ±0.617^a^ | 0.687 | ±0.222^a^ |
| 15 | 220 | 8 | 48.25 | ±17.36^a^ | 35.03 | ±8.73^a^ | 179.7 | ±23.66^a^ | 214.7 | ±22.45^ad^ | 1.888 | ±0.087^a^ | 0.641 | ±0.235^a^ |
| 15 | 220 | 24 | 67.73 | ±20.99^a^ | 30.37 | ±3.97^a^ | 177.3 | ±7.08^a^ | 207.7 | ±10.39 ^a^ | 2.053 | ±0.084^a^ | 0.929 | ±0.042^a^ |
| 22.5 | 331 | 0 | 54.44 | ±31.24^a^ | 36.53 | ±5.06^a^ | 120.7 | ±22.33^a^ | 157.2 | ±20.92^a^ | 2.129 | ±0.506^a^ | 0.843 | ±0.220^a^ |
| 22.5 | 331 | 2 | 68.95 | ±21.13^a^ | 52.11 | ±5.78^a^ | 181.1 | ±51.45^a^ | 233.3 | ±53.61^a^ | 2.733 | ±0.191^a^ | 1.004 | ±0.051^a^ |
| 22.5 | 331 | 4 | 84.54 | ±21.88^a^ | 53.21 | ±4.98^a^ | 258.4 | ±26.99^b^ | 311.6 | ±22.94^b^ | 2.661 | ±0.129^a^ | 0.891 | ±0.357^a^ |
| 22.5 | 331 | 8 | 75.15 | ±22.41^a^ | 31.98 | ±2.04^a^ | 258.4 | ±20.51^b^ | 290.4 | ±21.70^bc^ | 2.533 | ±0.199^b^ | 0.857 | ±0.397^a^ |
| 22.5 | 331 | 24 | 92.28 | ±24.94^a^ | 49.14 | ±21.36^b^ | 262.1 | ±42.09^b^ | 311.2 | ±58.49^b^ | 2.686 | ±0.229^b^ | 1.147 | ±0.066^ab^ |
| 30 | 442 | 0 | 54.44 | ±31.24^a^ | 36.53 | ±5.06^a^ | 120.7 | ±22.33^a^ | 157.2 | ±20.92^a^ | 2.129 | ±0.506^a^ | 0.843 | ±0.220^a^ |
| 30 | 442 | 2 | 54.58 | ±24.62^a^ | 50.09 | ±5.25^a^ | 169.4 | ±40.09^a^ | 219.5 | ±38.75^a^ | 2.656 | ±0.208^a^ | 1.034 | ±0.055^a^ |
| 30 | 442 | 4 | 87.48 | ±17.04^a^ | 51.40 | ±5.41^a^ | 284.3 | ±27.02^b^ | 335.7 | ±28.81^b^ | 2.739 | ±0.246^a^ | 0.611 | ±0.070^a^ |
| 30 | 442 | 8 | 90.25 | ±30.59^a^ | 39.81 | ±10.21^a^ | 247.8 | ±20.69^b^ | 287.6 | ±29.13^bc^ | 2.757 | ±0.357^b^ | 1.231 | ±0.160^b^ |
| 30 | 442 | 24 | 107.57 | ±29.20^a^ | 121.44 | ±25.62^c^ | 335.9 | ±82.73^c^ | 457.3 | ±87.25^c^ | 3.091 | ±0.350^b^ | 1.321 | ±0.132^b^ |
