# Supplementary material for: Manipulating the antioxidant capacity of halophytes to increase their cultural and economic value through saline cultivation
Source: AoB Plants. 2014 Aug 13;6:plu046. doi: 10.1093/aobpla/plu046 (PMC4174659; doi:10.1093/aobpla/plu046)
Supplement: Additional Information [file supp_plu046_plu046supp_file3.docx]

**File 3.** Mean values and SD (n=4) for *L. latifolium* dry matter (DM)*.* See Figure 1 for further details. Different letters at the same time point indicate significant differences (P≤0.05). All Pairwise Multiple Comparison Procedure (Holm Sidak Method) was applied. Abbreviations: Ascorbic acid, AA; catechin equivalents, CE; dehydroascorbic acid, DHA; gallic acid equivalents, GAE; total ascorbic acid, TAA; trolox equivalents, TE.

| **PSU** | **mM Na^+^ + Cl^-^** | **Time point** | **ORAC (µmol TE g^-1^ DM)** | | **AA (mg 100 g^-1^ DM)** | | **DHA (mg 100 g^-1^ DM)** | | **TAA (mg 100 g^-1^ DM)** | | **Total phenols (mg GAE g^-1^ DM)** | | **Total flavonoids (mg CE g^-1^ DM)** | |
| --- | --- | --- | --- | --- | --- | --- | --- | --- | --- | --- | --- | --- | --- | --- |
| 0 | 0 | 0 | 419.3 | ±250.8^a^ | 282.9 | ±59.9^a^ | 944 | ±281.1^a^ | 1227 | ±312.4^a^ | 16.55 | ±4.882^a^ | 6.534 | ±1.999^a^ |
| 0 | 0 | 2 | 587.9 | ±118.6^a^ | 294.0 | ±51.0^a^ | 1010 | ±88.2^a^ | 1304 | ±133.7^a^ | 16.92 | ±2.046^a^ | 6.566 | ±0.474^a^ |
| 0 | 0 | 4 | 305.5 | ±71.8^a^ | 280.2 | ±31.1^a^ | 1136 | ±288.1^a^ | 1416 | ±285.3^a^ | 14.81 | ±0.564^a^ | 5.876 | ±0.223^a^ |
| 0 | 0 | 8 | 385.2 | ±126.8^a^ | 277.7 | ±21.8^a^ | 1327 | ±77.8^a^ | 1604 | ±76.5^a^ | 13.37 | ±0.490^a^ | 4.742 | ±1.882^a^ |
| 0 | 0 | 24 | 463.9 | ±161.1^a^ | 204.3 | ±32.2^a^ | 1229 | ±29.2^a^ | 1434 | ±52.1^a^ | 14.76 | ±1.395^a^ | 7.001 | ±0.551^a^ |
| 15 | 220 | 0 | 419.3 | ±250.8^a^ | 282.9 | ±59.9^a^ | 944 | ±281.1^a^ | 1227 | ±312.4^a^ | 16.55 | ±4.882^a^ | 6.534 | ±1.999^a^ |
| 15 | 220 | 2 | 334.3 | ±170.0^a^ | 302.8 | ±100.0^a^ | 916 | ±231.2^a^ | 1219 | ±141.2^a^ | 16.36 | ±2.860^a^ | 5.878 | ±0.856^a^ |
| 15 | 220 | 4 | 482.0 | ±166.4^a^ | 293.2 | ±55.2^a^ | 1023 | ±364.6^a^ | 1317 | ±332.4^a^ | 15.30 | ±2.558^a^ | 4.485 | ±1.429^ab^ |
| 15 | 220 | 8 | 340.8 | ±115.7^a^ | 248.1 | ±55.4^a^ | 1281 | ±177.0^a^ | 1529 | ±149.7^a^ | 13.44 | ±0.277^a^ | 4.569 | ±1.695^a^ |
| 15 | 220 | 24 | 470.8 | ±124.6^a^ | 215.4 | ±39.2^a^ | 1252 | ±109.4^a^ | 1468 | ±147.3 ^a^ | 14.47 | ±0.504^a^ | 6.551 | ±0.398^a^ |
| 22.5 | 331 | 0 | 419.3 | ±250.8^a^ | 282.9 | ±59.9^a^ | 944 | ±281.1^a^ | 1227 | ±312.4^a^ | 16.55 | ±4.882^a^ | 6.534 | ±1.999^a^ |
| 22.5 | 331 | 2 | 393.5 | ±131.2^a^ | 295.0 | ±36.2^a^ | 1024 | ±294.5^a^ | 1319 | ±308.8^a^ | 15.48 | ±1.387^a^ | 5.681 | ±0.328^a^ |
| 22.5 | 331 | 4 | 511.6 | ±117.8^a^ | 330.5 | ±74.4^a^ | 1577 | ±148.2^b^ | 1908 | ±197.6^b^ | 16.33 | ±1.870^a^ | 5.499 | ±2.482^ab^ |
| 22.5 | 331 | 8 | 456.3 | ±128.8^a^ | 195.7 | ±18.4^a^ | 1575 | ±52.0^a^ | 1771 | ±67.7^a^ | 15.44 | ±0.387^a^ | 5.228 | ±2.418^a^ |
| 22.5 | 331 | 24 | 561.0 | ±125.0^a^ | 304.0 | ±143.5^a^ | 1610 | ±277.3^ab^ | 1914 | ±398.5^b^ | 16.44 | ±0.288^a^ | 7.058 | ±0.701^a^ |
| 30 | 442 | 0 | 419.3 | ±250.8^a^ | 282.9 | ±59.9^a^ | 944 | ±281.1^a^ | 1227 | ±312.4^a^ | 16.55 | ±4.882^a^ | 6.534 | ±1.999^a^ |
| 30 | 442 | 2 | 307.0 | ±137.0^a^ | 282.6 | ±32.0^a^ | 956 | ±233.4^a^ | 1239 | ±230.1^a^ | 14.96 | ±1.044^a^ | 5.825 | ±0.291^a^ |
| 30 | 442 | 4 | 432.4 | ±80.6^a^ | 254.2 | ±23.0^a^ | 1406 | ±123.4^ab^ | 1660 | ±126.3^ab^ | 13.55 | ±1.060^a^ | 3.022 | ±0.307^b^ |
| 30 | 442 | 8 | 481.9 | ±148.2^a^ | 212.6 | ±45.9^a^ | 1332 | ±61.2^a^ | 1544 | ±86.9^a^ | 14.79 | ±1.236^a^ | 6.601 | ±0.558^a^ |
| 30 | 442 | 24 | 571.0 | ±249.0^a^ | 654.6 | ±307.2^b^ | 1693 | ±199.3^b^ | 2348 | ±483.3 ^c^ | 15.96 | ±3.127^a^ | 6.815 | ±1.202^a^ |
