# Supplementary material for: Manipulating the antioxidant capacity of halophytes to increase their cultural and economic value through saline cultivation
Source: AoB Plants. 2014 Aug 13;6:plu046. doi: 10.1093/aobpla/plu046 (PMC4174659; doi:10.1093/aobpla/plu046)
Supplement: Additional Information [file supp_plu046_plu046supp_file4.docx]

| **Species** | **PSU** | **mM Na^+^ + Cl^-^** | **Glutathione**  **(nmol g^-1^ FM)** | | **Cysteine**  **(nmol g^-1^ FM)** | |
| --- | --- | --- | --- | --- | --- | --- |
| *T. pannonicum**^1^ | 15*^1^ | 220*^1^ | 29.5 | ±11.38 | 1.40 | ±0.29 |
| *T. pannonicum* | 15 | 220 | 55.6 | ±19.11 | 1.23 | ±0.13 |
| *T. pannonicum* | 22.5 | 331 | 58.8 | ±17.19 | 1.26 | ±0.14 |
| *T. pannonicum* | 30 | 442 | 80.4 | ±29.62 | 1.16 | ±0.07 |
| *T. pannonicum**^2^ | 15*^2^ | 220*^2^ | 18.5 |  | 1.44 |  |
| *T. pannonicum* | 15 | 220 | 43.8 | ±9.16 | 1.79 | ±0.64 |
| *S. dolichostachya**^2^ | 15*^2^ | 220*^2^ | 48.5 |  | 2.06 |  |
| *S. dolichostachya* | 15 | 220 | 42.2 | ±18.47 | 1.72 | ±0.27 |
| *P. coronopus**^2^ | 15*^2^ | 220*^2^ | 28.5 |  | 1.64 |  |
| *P. coronopus* | 15 | 220 | 23.1 | ±2.97 | 0.76 | ±0.15 |
| *L. latifolium**^2^ | 15*^2^ | 220*^2^ | 14.0 |  | 2.50 |  |
| *L. latifolium* | 15 | 220 | 169.1 | ±52.37 | 7.63 | ±0.92 |
| *A. portulacoides**^2^ | 15*^2^ | 220*^2^ | 208.1 |  | 16.96 |  |
| *A. portulacoides* | 15 | 220 | 126.5 | ±4.93 | 15.85 | ±2.93 |
| *A. halimus**^2^ | 15*^2^ | 220*^2^ | 278.2 |  | 24.45 |  |
| *A. halimus* | 15 | 220 | 155.9 | ±12.91 | 13.14 | ±2.39 |
| *B. cylindrica* | 15 | 220 | 122.5 | ±36.80 | 4.28 | ±2.16 |
| *B. cylindrica* | 30 | 331 | 142.1 | ±47.43 | 3.73 | ±1.26 |

**File 4.** Glutathione and cysteine concentrations of different species corresponding to different salinities. The asterisk marks the start values of younger plants *^1^ 4 weeks, *^2^ up to 7 weeks old. These plants were harvested just after the adaptation to 220 mM Na^+^ + Cl^-^/15 PSU.
